# DDX52 gene expression in LUAD tissues indicates potential as a prognostic biomarker and therapeutic target

**DOI:** 10.1038/s41598-023-44347-5

**Published:** 2023-10-13

**Authors:** Mingming Xu, Mingjun Yang

**Affiliations:** Department of Thoracic Surgery, Affiliated Hospital of Nantong University, 20 Xisi Street, Nantong, 226001 China

**Keywords:** Cancer, Biomarkers, Medical research, Molecular medicine, Oncology

## Abstract

Lung adenocarcinoma (LUAD) remains a leading cause of cancer-related morbidity and mortality globally. While DDX52, an ATP-dependent RNA helicase, plays a role in several biological processes, its specific involvement in LUAD is yet to be elucidated. We utilized ROC curves to determine DDX52’s predictive potential for LUAD. Kaplan–Meier survival curves, along with univariate and multivariate Cox analyses, assessed the prognostic implications of DDX52 in LUAD. We constructed nomogram models to further delineate DDX52’s influence on prognosis, employed GSEA for functional analysis, and used qRT-PCR to examine DDX52 expression in LUAD tissues. DDX52 expression was notably higher in LUAD tissues, suggesting its potential as a negative prognostic marker. We observed a direct relationship between DDX52 expression and advanced T and N stages, as well as higher grading and staging in LUAD patients. Cox analyses further underscored DDX52’s role as an independent prognostic determinant for LUAD. GSEA insights indicated DDX52’s influence on LUAD progression via multiple signaling pathways. Our nomogram, founded on DDX52 expression, effectively projected LUAD patient survival, as validated by calibration curves. Elevated DDX52 expression in LUAD tissues signals its potential as a poor prognostic marker. Our findings emphasize DDX52’s role not only as an independent prognostic factor for LUAD but also as a significant influencer in its progression through diverse signaling pathways. The constructed nomogram also underscores the feasibility of predicting LUAD patient survival based on DDX52 expression.

## Introduction

Lung cancer is a prominent malignancy, with lead being the predominant type, constituting approximately 40% of cases^1^. This subtype originates from alveolar and bronchiolar epithelial cells. These cells undergo malignant transformation marked by molecular changes, including gene mutations, abnormal protein expression, and alterations in signaling pathways^2^. Current medical focus is primarily on the early diagnosis and treatment of LUAD. Recent advancements in tumor molecular biology and genomics have led to the discovery and application of a plethora of molecular biomarkers. These biomarkers offer enhanced precision in diagnosing and assessing the prognosis of LUAD, while also opening up new therapeutic avenues^3^.

In the current landscape ofLUAD research, significant attention is centered on the Epidermal Growth Factor Receptor (EGFR), Kirsten Rat Sarcoma Viral Oncogene Homolog (KRAS), and Anaplastic Lymphoma Kinase (ALK) as pivotal molecular biomarkers. EGFR, a cell membrane receptor tyrosine kinase, is intricately associated with cell proliferation and metastasis in LUAD, making it a crucial treatment target^4^. The small GTPase KRAS, when mutated, can activate the MAPK and PI3K pathways, propelling tumor cell proliferation and invasion, despite a mutation frequency of up to 20%. Recent advancements suggest potential targeted therapies for certain KRAS mutations, particularly the G12C mutation. ALK pertains to a specific subtype of LUAD (specify the subtype for clarity). Compounds targeting ALK have shown promise in inhibiting the growth and metastasis of LUAD^5^. However, a significant proportion of lung cancer patients receive their diagnosis at advanced stages, limiting opportunities for surgical intervention. Many of these patients also exhibit reduced sensitivity to traditional chemotherapy and immunotherapy, leading to a markedly low 5-year survival rate^6,7^. This underscores the paramount importance of identifying novel and efficient molecular markers.

The DDX52 gene, encoding an RNA helicase, is acclaimed for its vital role in numerous biological processes, encompassing tumor growth, cell differentiation, ribosome biogenesis, and spermatogenesis. Its significance extends from its contribution to the onset and progression of various human ailments. For instance, studies on prostate cancer cells suggest that DDX52 inhibition can effectively retard cancer cell growth by modulating the c-Myc signaling pathway^8^. Findings from a zebrafish model emphasize DDX52’s role as a growth regulator during early developmental stages^9^. Similarly, targeting DDX52 has demonstrated suppression of melanoma cell proliferation through its influence on c-Myc^10^. Beyond its implications in cancer, DDX52 plays a crucial role in spermatogenesis. Specifically, the Es-DDX52 variant in the Chinese mitten crab (Eriocheir sinensis) is associated with spermatogonia mitosis and sperm cell differentiation^11^. In human lymphocytes, the expression and alternative splicing of the DDX52 gene significantly influence gene expression within the folate metabolism pathway^12^. Contemporary research has also spotlighted DDX52’s role in Human Immunodeficiency Virus 1 (HIV-1) replication^13^, and its effect on the cell specificity of the Myxoma virus in cancer therapy^14^. Comprehensive analyses have delved deep into DDX52’s architecture and function, notably its role in ribosome biogenesis^15^, and its structural comparison with other DEAD-box RNA helicases^16^.

In summary, the multifarious influence of the DDX52 gene on tumor growth, cell differentiation, ribosome biogenesis, and spermatogenesis warrants thorough investigation. This will not only deepen our understanding of these processes but also present potential targets for innovative therapeutic strategies.

## Materials and methods

### Data acquisition

We acquired normalized RNA-seq data and clinical information for LUAD patients from The Cancer Genome Atlas (TCGA) website (https://www.cancer.gov/ccg/research/genome-sequencing/tcga)^17^. Additionally, RNA-seq data for pan-cancer analysis and survival data were sourced from the XENA database, a platform offering comprehensive cancer genomic datasets (https://xenabrowser.net/datapages/)^18^.

### Quantitative real-time PCR (qRT-PCR)

For both LUAD tissue and adjacent normal tissue, we extracted total RNA using the TRIzol Reagent (Invitrogen). Subsequently, cDNA was synthesized using HiScript II (Vazyme, China), and qPCR analysis was conducted using the StepOne Plus Real-Time PCR system (Applied Biosystems, USA). The initial reaction was incubated at 95 °C for 10 min, followed by 40 cycles consisting of 95 °C for 15 s and 60 °C for 1 min. Relative expression levels were assessed using the 2-ΔCT method with the SYBR Green technique. All reactions were conducted in triplicate, using GAPDH as an internal reference^19^. The primers were synthesized by Sangon Biotech (Shanghai) Co., Ltd. In this study, we collected tumor tissues and adjacent normal tissues from 8 LUAD patients at the affiliated hospital of Nantong University. The research was approved by the Ethics Committee of the affiliated hospital of Nantong University. We strictly adhered to the guidelines set by China’s “Regulations on the Implementation of the Human Genetic Resources Management” and obtained informed consent from all participants (NO.2022-K046). The primer sequences are as follows: GAPDH Forward: TGCACCACAACTGCTTAGC; GAPDH Reverse: GGCATGGACTGTGGTCATGAG; DDX52 Forward: CTTCTGGCTTCTGCTCCAACTGG; DDX52 Reverse: TGTGAATCTGGCTGGCAAGTTCTC.

### Univariate and multivariate Cox regression analyses and ROC analysis

Both univariate and multivariate Cox regression analyses were performed to identify independent prognostic factors influencing Overall Survival (OS), Disease-Specific Survival (DSS), Progression-Free Interval (PFI), and Disease-Free Interval (DFI) in LUAD patients. Covariates considered included age, gender, stage, T stage, N stage, M stage, and the DDX52 gene. To assess the diagnostic performance of the DDX52 gene, we used the Xiantao Academic database, a platform for clinical diagnostic metrics (https://www.xiantaozi.com/)^20^, and calculated the area under the ROC curve. We also employed the R package “timeROC” to determine the ROC curve area under the curve for DDX52 in LUAD patients at 1 year, 5 years, and 10 years.

### Construction of a predictive prognostic column chart

To predict survival probabilities at 1 year, 3 years, and 5 years, we used the R package “rms” to visualize the relationship between overall survival rates and predictive factors, including age, gender, stage, T stage, M stage, N stage, and the DDX52 gene. In the column chart, a point proportion scale was employed to assign points to each parameter, with these being summed for an overall point score^21^.

### Gene set enrichment analysis (GSEA)

We obtained the GSEA software from its official website (https://www.gsea-msigdb.org/gsea/index.jsp) and prepared the required files as per the provided instructions. For stratifying the LUAD samples in our study, the median expression of the DDX52 gene was used, leading to the division of samples into two distinct groups. These files were then imported into the GSEA software. Our analysis primarily focused on the gene set “c2.cp.kegg.v7.5.1.symbols.gmt”. We ran 1000 permutation experiments, identifying pathways of significant biological relevance with P-values < 0.05. Based on these criteria, we highlighted pathways exhibiting substantial biological significance^22^.

### Statistical analyses

Statistical analyses and chart creation were done using R 4.1.3 (https://www.r-project.org/) and GraphPad Prism 8.0. The relationship between DDX52 and clinical-pathological parameters was explored using the Wilcoxon and Kruskal–Wallis tests. The Kaplan–Meier curve was employed to evaluate the predictive capacity of the DDX52 gene for LUAD patient survival. Univariate and multivariate Cox regression analyses were used to examine associations between variables and OS, DFI, PFI, and DSS. A column chart was generated with the rms package in R software, setting statistical significance at P < 0.05.

### Ethics statement

In this study, tumor tissues and adjacent normal tissues were collected from 8 LUAD patients at the Affiliated Hospital of Nantong University. This study was approved by the Ethics Committee of Affiliated Hospital of Nantong University. We strictly follow the guidelines of China’s Implementing Regulations on the Management of Human Genetic Resources and have obtained the informed consent of all participants (No. 2022-K046).

## Results

### Prognostic analysis of DEAD-box helicases family genes in LUAD

**Figure 1:**
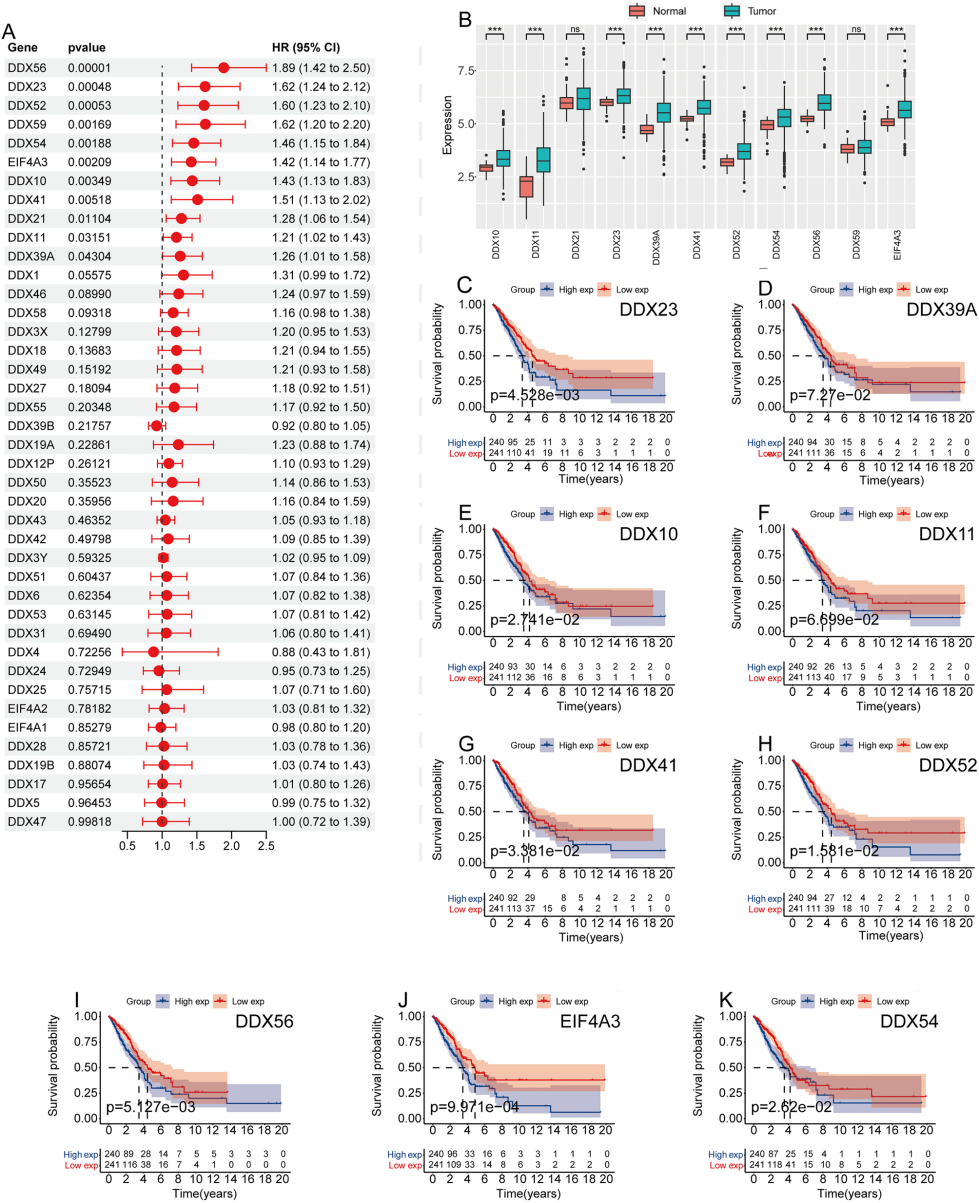
Prognostic analysis of DEAD-box helicases family genes in LUAD. (**A**) Univariate Cox analysis of the DEAD-box helicases family genes. (**B**) Box plots depict the expression of 11 genes, including DDX56, DDX23, DDX52, DDX59, DDX54, EIF4A3, DDX10, DDX41, DDX21, DDX11, and DDX39, in normal samples and LUAD samples from the TCGA database. (**C**–**K**) Kaplan–Meier survival curves assess the prognostic impact of these genes including DDX56, DDX23, DDX52, DDX54, EIF4A3, DDX10, DDX41, DDX11, and DDX39 on LUAD.

First, we conducted a univariate Cox analysis on the DEAD-box helicases family genes within the TCGA LUAD dataset. We identified DDX56, DDX23, DDX52, DDX59, DDX54, EIF4A3, DDX10, DDX41, DDX21, DDX11, and DDX39A as potential genes impacting LUAD prognosis (Fig. 1A). We then visualized the expression levels of these 11 genes using box plots. We found that, apart from DDX21 and DDX59, the expression of the remaining genes was notably elevated in LUAD (Fig. 1B). Survival analysis using Kaplan–Meier curves for the 9 differentially expressed DEAD-box helicases family genes revealed that elevated expression of seven genes—DDX56, DDX23, DDX52, DDX54, EIF4A3, DDX10, and DDX41—was indicative of poor prognosis in LUAD patients (Fig. 1C–K). This underscores the influence of DEAD-box helicases family genes on the prognosis of LUAD.

### DDX52 gene as a poor prognostic indicator for LUAD patients

**Figure 2:**
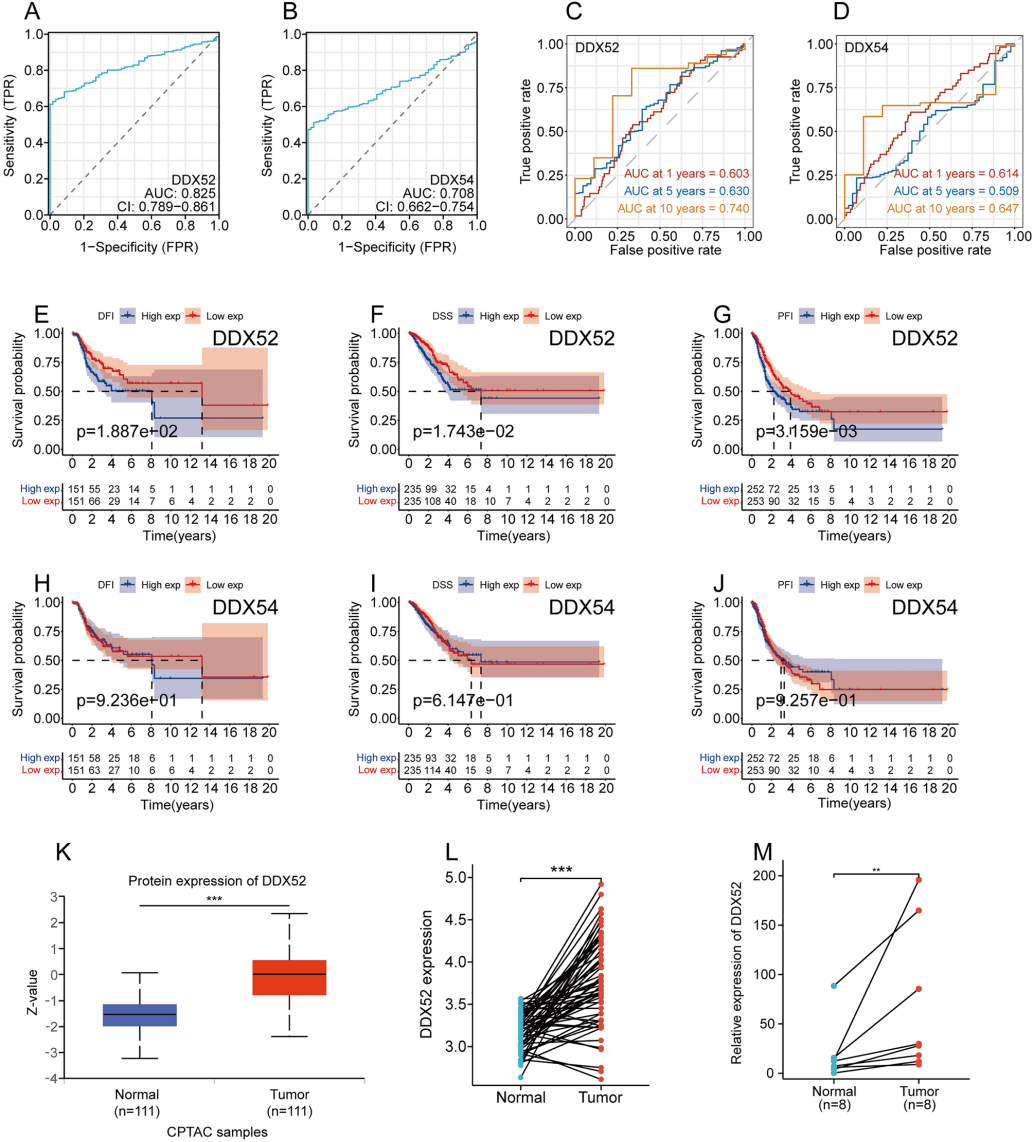
DDX52 gene as a poor prognostic indicator for LUAD patients. (**A**,**B**) Diagnostic ROC curves for DDX52 and DDX54 genes. (**C**,**D**) Time-dependent ROC curves for DDX52 and DDX54 genes. (**E**–**J**) Kaplan–Meier survival curves analyzing the effect of DDX52 and DDX54 genes on LUAD DSS, DFS, and PFI. (**K**) DDX52 protein expression analysis in LUAD and normal lung tissue from the CPTAC database. (**L**) Expression of DDX52 in paired LUAD samples versus normal lung samples. (**M**) qRT-PCR validation of DDX52 gene expression in 8 paired LUAD tissues and their corresponding normal tissues.

While genes DDX56, DDX23, EIF4A3, DDX10, and DDX41 have been previously studied in LUAD, the roles of DDX52 and DDX54 remain unexplored. Further analysis of DDX52 and DDX54 in the TCGA LUAD dataset using diagnostic ROC curves revealed their significant diagnostic value, with AUCs of 0.825 and 0.708, respectively (Fig. 2A,B). Subsequent time-dependent ROC curve analysis showed that DDX52 had superior diagnostic potential in later stages, with 1-year, 5-year, and 10-year AUCs of 0.603, 0.630, and 0.740, respectively. In contrast, DDX54’s corresponding AUCs were 0.614, 0.509, and 0.647 (Fig. 2C,D). Additionally, Kaplan–Meier survival curve analysis indicated that high expression of DDX52 correlates with poor DFI, DSS, and PFI outcomes (Fig. 2E–G). In contrast, DDX54 expression showed no significant correlation with LUAD’s DFI, DSS, and PFI (Fig. 2H–J). Hence, DDX52 appears to hold greater research potential. Analysis from the CPTAC database indicated that DDX52 protein expression is elevated in LUAD tissue compared to normal lung tissue (Fig. 2K). Similarly, in the TCGA LUAD dataset, a comparison between paired adenocarcinoma lung samples and normal lung tissue samples revealed a notable upregulation of DDX52 gene expression in the adenocarcinoma samples (Fig. 2L). Moreover, qRT-PCR analysis of 8 paired lung cancer tissues and their corresponding normal lung tissues confirmed the increased expression of DDX52 in lung cancer tissues (Fig. 1M).

### Correlation of DDX52 gene expression with clinical characteristics

**Figure 3:**
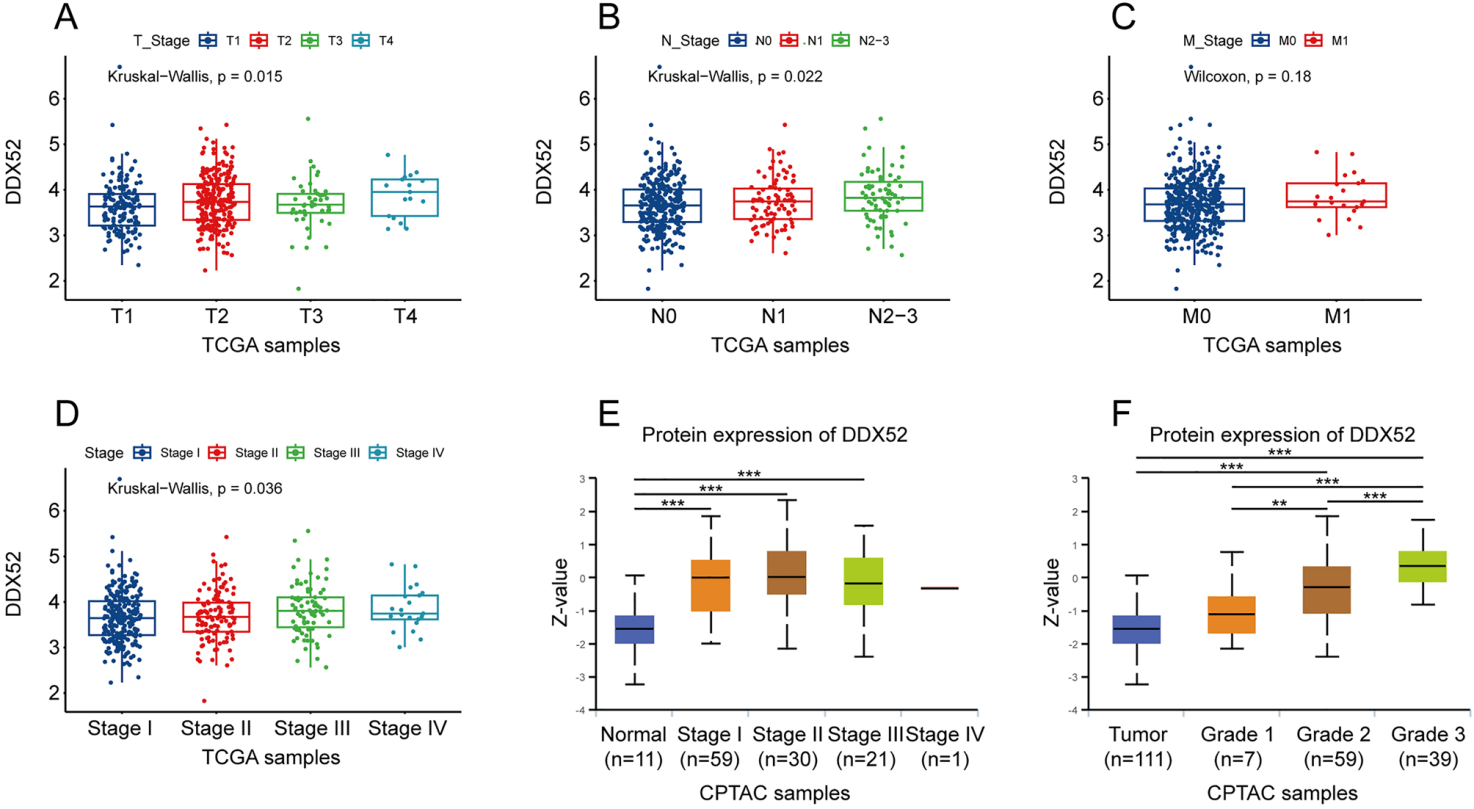
Correlation of DDX52 gene expression with clinical characteristics. (**A**,**B**) Positive correlation between DDX52 expression and both T stage and N stage of LUAD patients in the TCGA database. (**C**) No marked difference in DDX52 expression between M0 and M1 stage LUAD patients from the TCGA database. (**D**,**E**) Analysis of DDX52 expression correlated with stages and grades of LUAD patients from TCGA and CPTAC databases.

We delved into the relationship between DDX52 gene expression and clinical characteristics of LUAD patients. In the TCGA database, a positive correlation emerged between DDX52 gene expression and T and N stages (Fig. 3A,B). Although no significant difference in DDX52 gene expression was noted between M0 and M1 stage patients (Fig. 3C), its expression consistently increased with higher Stage (Fig. 3D). Similarly, analyzing the CPTAC database unveiled an increased expression of the DDX52 gene with higher Grade, even though no difference was found among Stage I-VI classifications (Fig. 3E,F).

### Independent prognostic role of DDX52 gene in LUAD patients

**Figure 4:**
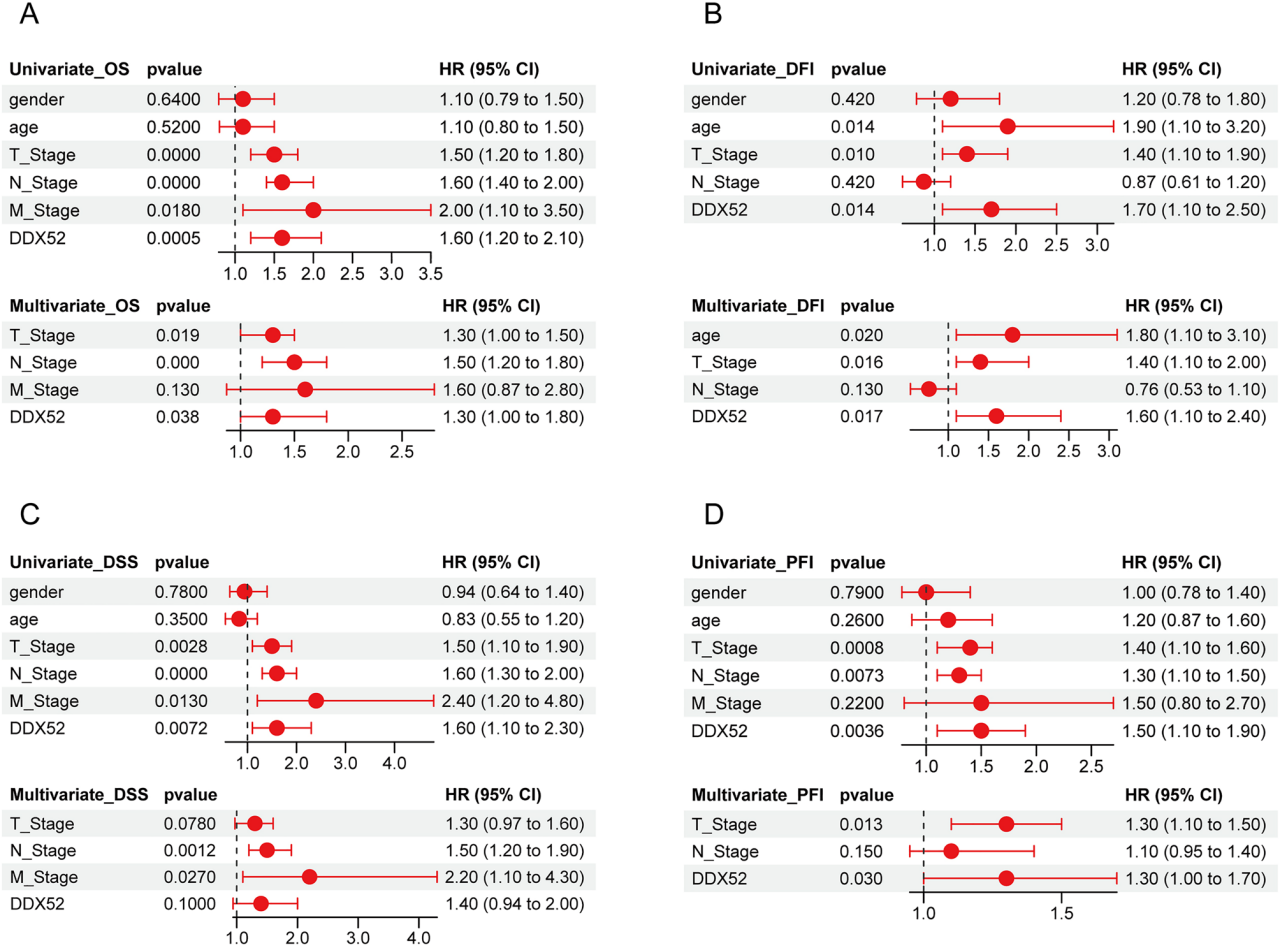
Independent prognostic role of DDX52 gene in LUAD patients. (**A**–**D**) Univariate and multivariate Cox regression analyses for various prognostic measures of LUAD patients, highlighting the independent significance of DDX52 gene in OS, DFI, DSS, and PFI prognosis.

To unravel the influence of the DDX52 gene on LUAD patient prognosis, we conducted univariate and multivariate Cox analyses, considering factors such as gender, age, T stage, N stage, M stage, and DDX52 gene expression. Results confirmed that T stage, N stage, and DDX52 gene expression independently affected OS prognosis in LUAD patients (Fig. 4A). For DFI prognosis, age, T stage, and DDX52 gene expression were identified as independent prognostic factors (Fig. 4B). N stage and M stage emerged as independent prognostic factors for DSS prognosis (Fig. 4C). Furthermore, T stage and DDX52 gene expression stood out as independent prognostic factors for PFI prognosis (Fig. 4D). While not independently impacting DSS, the DDX52 gene’s significance in LUAD patient prognosis was evident.

### GSEA analysis

**Figure 5:**
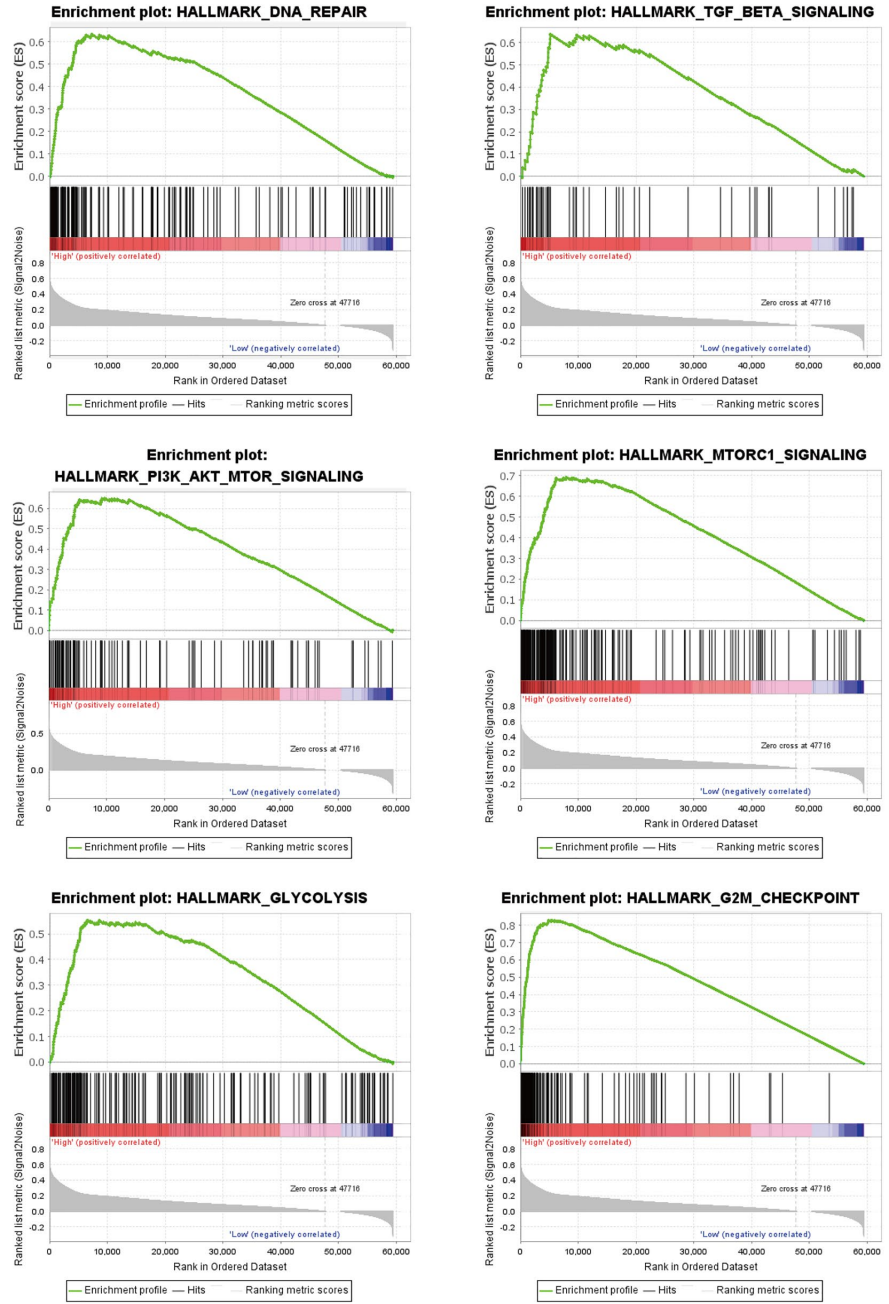
GSEA analysis. (**A**) GSEA reveals potential pathways regulated by the DDX52 gene, influencing LUAD progression.

In-depth exploration of the DDX52 gene’s role in LUAD involved GSEA analysis, revealing its potential influence on pathways like DNA_REPAIR, G2M_CHECKPOINT, GLYCOLYSIS, MTORC1_SIGNALING, PI3K_AKT_MTOR_SIGNALING, and TGF_BETA_SIGNALING (Fig. 5A).

### Predictive potential of the DDX52 gene through nomogram model

**Figure 6:**
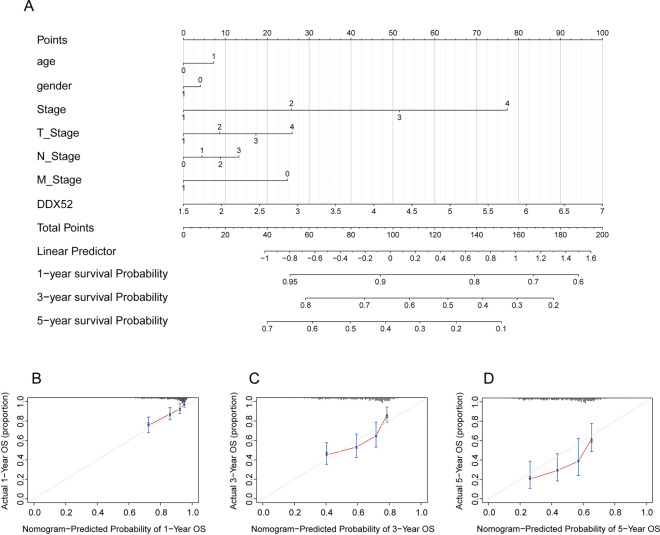
Predictive potential of the DDX52 gene through nomogram model. (**A**) Nomogram predicts LUAD patient survival at 1, 3, and 5 years based on DDX52 expression. (**B**–**D**) Calibration curves validate the model’s accuracy in LUAD prognosis prediction.

In a bid to enhance the predictive capacity of the DDX52 gene for LUAD patient prognosis, we introduced a Nomogram model. This model effectively gauged the 1-, 3-, and 5-year survival of LUAD patients based on DDX52 gene expression levels (Fig. 6A). Calibration curves substantiated the model’s reliability in prognostication (Fig. 6B–D).

### DDX52 gene expression in pan-cancer context

**Figure 7:**
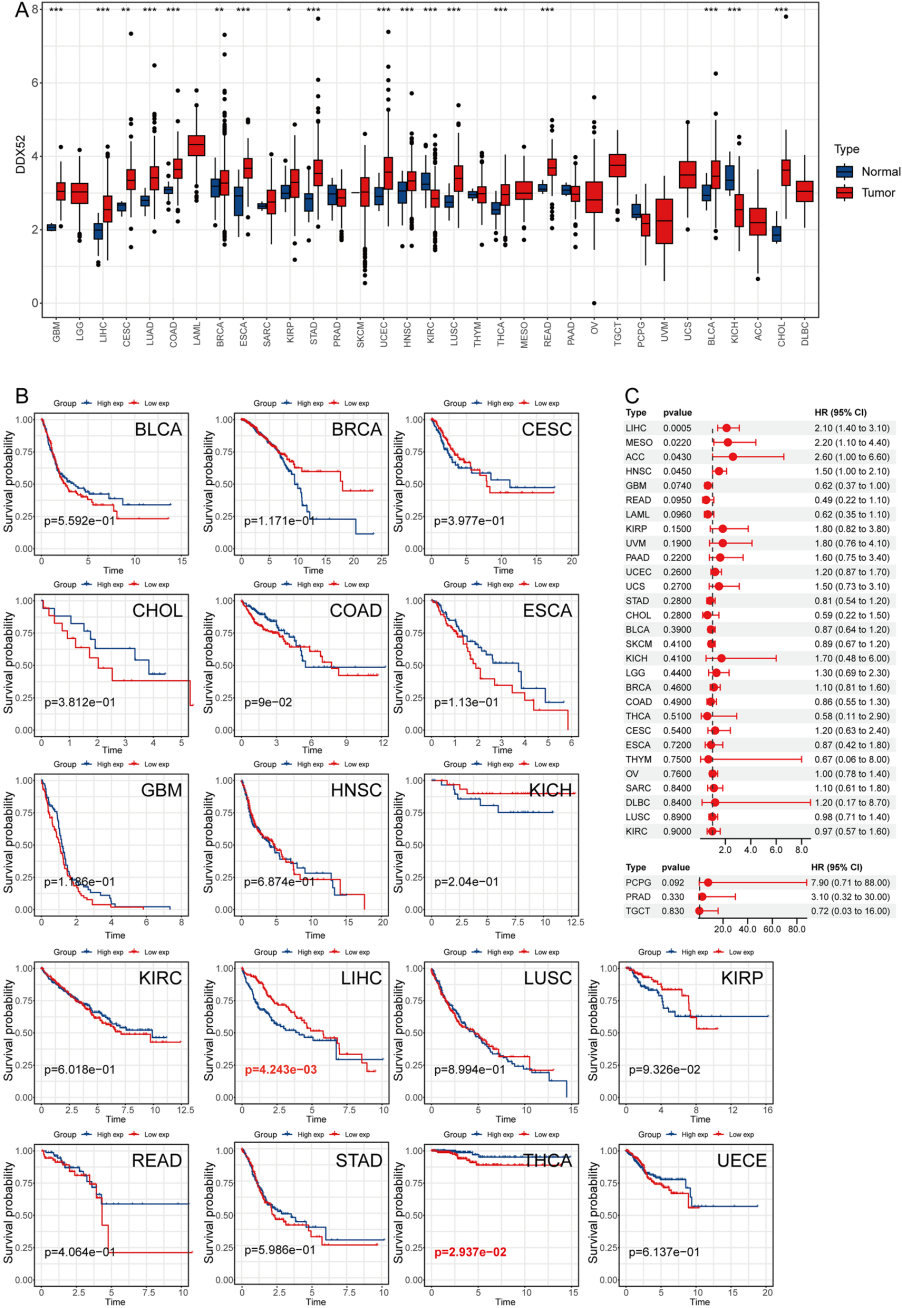
DDX52 gene expression in pan-cancer context. (**A**) Expression pattern of DDX52 gene across various cancers from TCGA data. (**B**,**C**) Kaplan–Meier survival curve analysis and univariate Cox analysis of the DDX52 gene in different tumors.

Broadening our perspective to pan-cancer data, we examined the DDX52 gene’s expression across various cancers. Our findings highlighted its abnormal elevation in GBM, LICH, CESC, COAD, BRCA, ESCA, KIRP, STAD, UCEC, HNSC, LUSC, THCA, READ, BLCA, and CHOL, whereas it was suppressed in KIRC and KICH (Fig. 7A). Kaplan–Meier survival curve analysis unveiled a correlation between high DDX52 gene expression and poor prognosis in LICH and THCA (Fig. 7B). Univariate Cox analysis identified the DDX52 gene as a prognostic factor in LICH, MESO, ACC, and HNSC (Fig. 7C). These findings underscore the DDX52 gene’s aberrant expression across LUAD and several other cancers, associating its high expression with unfavorable prognosis. Consequently, the DDX52 gene emerges as a potential therapeutic and prognostic marker, with substantial implications for cancer diagnosis and treatment.

## Discussion

Lung cancer, particularly its most common subtype, LUAD, is a predominant malignant tumor worldwide^23^. While advances have been made in LUAD treatment, patient prognosis remains challenging. This necessitates an in-depth exploration into LUAD’s pathogenesis and the development of innovative treatment strategies. A strong association has been found between dysregulated regulatory genes and cancer progression.

The DEAD-box helicases family represents a class of RNA helicases, characterized by the conserved Asp-Glu-Ala-Asp (DEAD) motif. Integral to multiple RNA metabolism stages, these helicases partake in processes like RNA splicing, ribosome assembly, translation initiation, and RNA decay. An increasing body of evidence links DEAD-box helicases to cancer progression. Altered expression and activity of these genes are observed across various malignancies, impacting cancer cell behaviors such as proliferation and migration. For instance, DDX10’s high expression in colorectal cancer tissues suggests its potential as a diagnostic and therapeutic marker^24^. Similarly, suppressing the overexpressed DDX56 inhibits gastric cancer growth, indicating its therapeutic potential^25^. Using the TCGA LUAD dataset, we examined the influence of DEAD-box helicase family genes on LUAD. Seven genes—DDX56, DDX23, DDX52, DDX54, EIF4A3, DDX10, and DDX41—demonstrated significant upregulation in LUAD samples, suggesting their potential as LUAD prognostic indicators. For example, DDX56’s modulation of Wnt signaling genes indicates a possible link to early relapse in squamous cell lung cancer^26^. EIF4A3’s upregulation in LUAD associates with poor prognosis, possibly due to interactions impacting LUAD cell behaviors^27^. Additionally, APE1’s interaction with AIM2 and DDX41 highlights its therapeutic potential in addressing radiation resistance and thermotolerance^28^. In-depth analysis on DDX52 and DDX54 revealed DDX52’s superior diagnostic capabilities for LUAD. Survival analysis further highlighted DDX52’s prognostic potential for LUAD patients. Despite its dysregulation across various tumors, comprehensive studies on DDX52’s expression and prognostic significance in LUAD are limited. Our combined analysis of the TCGA and CPTAC databases, along with qRT-PCR experiments, showcased the elevated expression of the DDX52 gene in LUAD compared to normal lung tissues. Importantly, DDX52 gene expression correlates with T and N staging, as well as overall stage, advocating for its potential as a LUAD prognostic biomarker.

Investigating the regulatory mechanisms underlying DDX52 gene involvement in lung cancer progression, we conducted an in-depth analysis utilizing GSEA. The outcomes revealed substantial enrichment of the DDX52 gene across various pathways, notably the cell cycle, DNA replication, ERBB, MAPK, mTOR, cancer pathways, TGF-beta, and Wnt. Of particular significance, the cell cycle pathway stands as a critical player in lung cancer development and progression. It encompasses dysregulated expression of cell cycle regulatory proteins and pivotal tumor suppressor genes, such as p53. Notably, mutations in these genes have been closely linked to the advancement of lung cancer^29^. Likewise, the aberrant control of DNA replication emerges as a hallmark of cancer. Genes associated with DNA replication, including RECQL4, have demonstrated a clear connection to lung cancer^30^. The ERBB pathway assumes a vital role in the landscape of lung cancer development and progression, exerting control over cell proliferation, survival, and differentiation. Noteworthy is the connection between overexpression and activation of the ERBB receptor family and the pathogenesis of non-small cell lung cancer^31^. In parallel, the MAPK pathway emerges as a key player in lung cancer advancement. Its activation significantly boosts lung cancer cell proliferation and survival, thereby correlating with unfavorable prognosis among NSCLC patients^32^. Similarly, the mTOR pathway, governing cell growth and metabolism, exhibits critical dysregulation linked to lung cancer pathogenesis. Notably, its activation strongly associates with poor prognosis among NSCLC patients^33^. Pathways in Cancer, a repository of pivotal signaling pathways in malignancies, has spotlighted the integral roles of pathways like ERBB, MAPK, and mTOR in lung cancer pathogenesis. Meanwhile, the TGF-beta pathway governs physiological processes within lung cancer cells, dictating proliferation, differentiation, and apoptosis. Perturbation of this pathway closely associates with NSCLC pathogenesis^34^. Equally, Wnt pathway dysregulation surfaces as a significant contributor to lung cancer development and progression, accentuated by its promotion of lung cancer cell survival and proliferation^35^. Collectively, these intricate pathways likely interplay to orchestrate the regulatory mechanism of the DDX52 gene in the context of lung cancer.

Overall, these findings indicate a potential significant role of the DDX52 gene in the onset and progression of LUAD. Consequently, the DDX52 gene holds promise as both a potential therapeutic target and a prognostic marker for individuals with LUAD.

## Conclusions

In summary, our investigation clearly demonstrates the significant upregulation of the DDX52 gene in LUAD tissues, establishing it as a negative prognostic indicator for LUAD patients. Notably, elevated expression of the DDX52 gene shows a positive correlation with T and N stages, exhibiting a concurrent increase with tumor stage and grade. Furthermore, our analysis establishes the DDX52 gene’s independence as a prognostic factor for LUAD patients, with its predictive accuracy effectively represented by the Nomogram model. Through GSEA analysis, we have unveiled potential pathways by which the DDX52 gene may regulate LUAD progression, including Cell_Cycle, DNA_Replication, ERBB_Signaling_Pathway, MAPK_Signaling_Pathway, mTOR_Signaling_Pathway, Pathways_in_Cancer, TGF_Beta_Signaling_Pathway, Ubiquitin_Mediated_Proteolysis, and Wnt_Signaling_Pathway. Moreover, our pan-cancer investigation highlights the aberrant elevation of the DDX52 gene across various other cancer types. Collectively, these findings underscore the therapeutic potential of targeting the DDX52 gene not only for LUAD but also for diverse cancers.

## Data Availability

The raw data used in our analyses can be downloaded from the TCGA database (https://portal.gdc.cancer.gov/). “h.all.v7.5.1.symbols.gmt” dataset was downloaded from the GSEA database (https://www.gsea-msigdb.org/gsea/index.jsp). The datasets used or analysed in this study are available from the corresponding authors on request.
